# Percutaneous transmitral balloon commissurotomy using a single balloon with arteriovenous loop stabilisation: an alternative when there is no Inoue balloon

**DOI:** 10.5830/CVJA-2018-010

**Published:** 2018

**Authors:** Tefera Endale, Leye Mohamed, Miró Joaquim, Garceau Patrick, Bouchard Denis

**Affiliations:** Department of Paediatrics and Child Health, Cardiology Division, School of Medicine, Addis Ababa University, Addis Ababa, Ethiopia; Division of Paediatric Cardiology, CHU Sainte-Justine, Université de Montréal, QC, Canada; Division of Paediatric Cardiology, CHU Sainte-Justine, Université de Montréal, QC, Canada; Department of Medicine, Montréal Heart Institute, Université de Montréal, QC, Canada; Division of Cardiovascular Surgery, Montreal Institute of Cardiology, Université de Montréal, QC, Canada

**Keywords:** arteriovenous loop stabilisation, balloon mitral commissurotomy, modified Nucleus balloon technique, mitral valvotomy in resource-limited settings

## Abstract

**Background:**

The Inoue balloon technique is the standard technique for mitral valve balloon commissurotomy at this stage. However, the hardware for this technique is expensive and may not always be available in resource-limited settings.

**Objectives:**

This article reports our experience with percutaneous transmitral balloon commissurotomy using a single balloon (Nucleus) with arteriovenous loop stabilisation.

**Methods:**

Eleven young patients, aged 12–26 years and weighing 23–48 kg, underwent transmitral balloon commissurotomy using the described technique at our centre from April to May 2014.

**Results:**

Mean fluoroscopy time was 22.6 ± 6.4 min (18.5– 30.0). Mean transmitral gradient decreased from 24.1 ± 5.9 (16–35) to 6.6 ± 3.8 (3–14) mmHg, as measured on transoesophageal echocardiography. Mean mitral valve area increased from 0.69 ± 0.13 cm^2^ (range 0.5–0.9) before dilation to 1.44 ± 0.25 cm^2^ (1.1–1.9) after dilation (p < 0.001). Mean estimated pulmonary artery systolic pressure decreased from 110.0 ± 35 mmHg (75–170) before dilation to 28.0 ± 14.4 mmHg (range 10–60) after dilation.

**Conclusion:**

Our modified Nucleus balloon technique for mitral valve dilation in young patients with mitral stenosis is effective and safe. The technique differs from other over-thewire techniques in that it avoids placing stiff wire in the left ventricle. It also offers better balloon stability and control owing to the arteriovenous loop. This technique may be easier for use by paediatric interventionists who might not be familiar with the Inoue balloon technique.

Although it has become exceedingly rare in the developed world, rheumatic heart disease continues to be a serious health problem in developing nations.^1^ Unlike other valvular lesions, which might be attributed to multiple aetiologies, mitral stenosis alone or in combination with other valvular lesions is the only lesion almost exclusively attributed to rheumatic heart disease.^2^,^3^ Studies from developing countries have shown that mitral stenosis progresses rapidly and may lead to serious disability early in life.^4^-^7^ Commissural fusion, leaflet thickening and alteration of the subvalvular apparatus are the dominant mechanisms causing clinically important mitral stenosis of rheumatic origin.^8^

As mitral stenosis is a mechanical obstruction to forward flow, the only definitive treatment is mechanical relief of the obstruction. Such invasive treatments include closed mitral commissurotomy, open mitral valve repair, mitral valve replacement, or percutaneous transmitral balloon commissurotomy.^9^-^11^ Percutaneous transmitral commissurotomy is associated with significant changes in mitral valve morphology in terms of splitting of the fused mitral commissures, increased mitral valve area, improved leaflet excursion and splitting of the sub-valvular structures.^12^

A variety of hardware and techniques has been described. These include the Inoue balloon technique, single-balloon over-the-wire techniques, double-balloon technique, multi-track system, metallic valvotome and other similar techniques.^13^-^17^ Currently, the Inoue balloon is the standard technique. However, the hardware for this technique is expensive and may not always be available in resource-limited settings. In this article, we describe a technique for balloon mitral commissurotomy using a single Nucleus balloon, with arteriovenous loop stabilisation.

## Methods

This technique is a modification of the regular single-balloon, over-the-wire technique described previously,^18^ and adapted by subsequent workers.^19^ It was modified according to the available materials at our centre during that period, and to adapt to the relatively small size of our patients.

A total of 11 patients, all teenagers or young adults, underwent transmitral balloon commissurotomy using the described technique in our centre from April to May 2014. Eight patients (72.7%) were female. In all patients, diagnosis of mitral stenosis was made on the first presentation to medical attention.

Mean mitral valve area, measured by planimetry on transoesophageal echocardiography (TEE), was 0.69 ± 0.13 cm^2^ (range 0.5–0.9). Mean transmitral diastolic gradient was 24.1 ± 5.9 mmHg (range 16–35) and mean estimated pulmonary artery systolic pressure was 110.0 ± 35 mmHg (75–170). Other baseline characteristics of the patients are shown in Table 1.

**Table 1:** Baseline characteristics of patients treated for severe rheumatic mitral stenosis using a modified Nucleus balloon technique

*Variables*	*Mean ± SD (range)*
Age (years)	14.3 ± 4.2 (12–26)
Weight (kg)	30.3 ± 7.4 (23–48)
Height (cm)	146.6 ± 9.9 (133–163)
Spontaneous echo contrast in the left atrium (number of patients)	5
NYHA functional class	
Class I	
Class II	3
Class III	8
Class IV	

Under general anaesthesia, right femoral vein access was taken with a 7F short sheath. A 0.025-inch regular wire was advanced up the superior caval vein and a 7F long sheath was advanced to the left innominate vein. The wire was withdrawn and a Brockenbrough needle (Medtronic Inc, Minneapolis, MN, USA) was introduced.

After sliding the system down to the oval fossa, transseptal puncture was performed under TEE and single-plane fluoroscopy guidance. The needle was withdrawn into the dilator and the sheath–dilator assembly was advanced into the left atrium. Once in the left atrium, the dilator was removed.

**Fig. 1:**
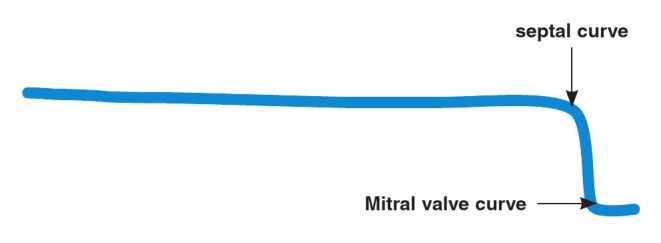
Pre-shaped stiff end of a regular guide wire with septal and mitral valve cuves for guiding the wedge balloon across the mitral valve

A 6F 110-cm-long wedge balloon catheter (Arrow Int, Inc, Bernville Rd, Reading PA, USA) was advanced into the left atrium. The pre-formed stiff end of a regular wire (Fig. 1) was used as a stylet to guide the inflated wedge balloon across the mitral valve. Once the balloon was at the apex of the left ventricle, its position at the centre of the mitral apparatus (and not through the chordae) was confirmed by TEE.

**Fig. 2:**
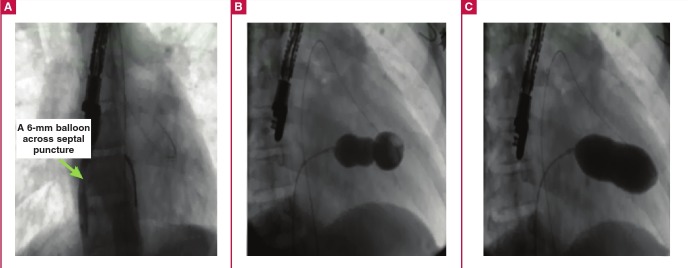
A. Establishment of arteriovenous loop and dilation of the septal puncture. B. Nucleus balloon inflated across a severely stenotic mitral valve. C. Full inflation of the Nucleus balloon across the mitral valve showing near-disappearance of the waist.

The wedge balloon catheter was then advanced up the ascending aorta, using the pre-shaped end of the stiff wire, if necessary. A 0.035-in × 260-cm Terumo wire (Terumo Medical Corporation, Cottontail Lane, Somerset, New Jersey, USA) was advanced through the wedge catheter and the wire was snared to the descending aorta from the arterial side to establish an arteriovenous loop (Fig. 2A). Then the inflated wedge catheter was again withdrawn into the left ventricle and pushed and pulled gently through the mitral valve apparatus to ascertain that there was no entrapment within the mitral valve chordae. The wedge balloon and the long sheath were then removed.

A 12F to 14F short sheath was introduced into the femoral vein. The septal puncture was dilated with a 6- or 8-mm balloon (Fig. 2A). Finally, a Nucleus balloon (NuMED Canada Inc, Second Street West Cornwall, ON, Canada) of appropriate size, according to the patient’s size and TEE measurement of the mitral annulus, was introduced and placed across the mitral valve. We did not encounter any difficulty with passing the balloon through the septal puncture in any of our patients.

A 20-cm^3^ syringe with 25% contrast and 75% saline combination was attached using a three-way stopcock. An inflation device filled with a similar combination of contrast and saline was attached. The desired inflation pressure was decided based on the table provided with the balloon (Table 2), in order to achieve the exact target diameter. Both ends of the arteriovenous loop were pulled to stabilise the balloon in a good position and the balloon was inflated using fluoroscopic and TEE guidance.

**Table 2:** Nominal balloon diameter versus inflation pressures and corresponding effective balloon diameters obtained

	*Balloon diameters*					
*Applied pressure*	*18.0 mm*	*20.0 mm*	*22.0 mm*	*25.0 mm*	*28.0 mm*	*30.0 mm*
1 atm	15.5	16.7	19.0	21.8	24.4	25.9
2 atm	16.1	17.3	19.6	22.9	27.4	29.7
3 atm	16.9	17.3	19.6	22.9		
4 atm	17.9	19.9				

Maximum precautions were taken not to inflate the balloon in the left ventricular outflow tract. Inflation was first with the syringe and then with the inflation device, since the total volume frequently exceeded the capacity of the inflation device. A waist formed and then disappeared (Fig. 2B, C). Inflations at increased pressure were repeated if needed, with control for degree of mitral regurgitation, mean transmitral diastolic gradient and post-dilation mitral valve area on TEE.

## Results

Mean fluoroscopy time was 22.6 ± 6.4 min (18.5–30.0). Mean transmitral gradient decreased from 24.1 ± 5.9 (16–35) to 6.6 ± 3.8 (3–14) mmHg, as measured on TEE. Mean mitral valve area increased from 0.69 ± 0.13 cm^2^ (range 0.5–0.9) before dilation to 1.44 ± 0.25 cm^2^ (range 1.1–1.9) after dilation (p < .001). Mean estimated pulmonary artery systolic pressure decreased from 110.0 ± 35 mmHg (range 75–170) before dilation to 28.0 ± 14.4 mmHg (range 10–60) immediately after dilation on TEE. Outcome variables after balloon dilation are shown in Table 3.

**Table 3:** Outcome variables in patients treated for severe rheumatic mitral stenosis using the modified Nucleus balloon technique

*Variables*	*Before dilation mean ± SD (range)*	*After dilation mean ± SD (range)*	*p-value*
Mitral valve area (cm^2^) by Planimetry	0.69 ± 0.13 (0.5–0.9)	1.44 ± 0.25 (1.1–1.9)	< 0.001
Mean transmitral gradient (mmHg)	24.1 ± 5.9 (16–35)	6.6 ± 3.8 (3–14)	< 0.001
Average estimated pulmonary artery systolic pressure (mmHg)	110.0 ± 35 (75–170)	28.0 ± 14.4 (10–60)	< 0.001
Mitral regurgitation			
Severe	-	1	
Moderate	-	1	
Mild	2	5	
Trivial	5	2	
None	4	1	
Tricuspid regurgitation			
Severe	-	-	
Moderate	1		
Mild	6	6	
Trivial	4	3	
None	-	1	

One patient developed severe mitral regurgitation due to a tear on the anterior mitral valve leaflet and she underwent semiurgent valve replacement surgery. Another patient developed moderate mitral regurgitation, which was well tolerated. No complications were noted in the other patients either immediately after the procedure or on subsequent follow up.

At the follow up, up to 20 months later, all the patients were in NYHA functional class I–II. Mean mitral valve area remained stable at 1.43 ± 0.32 cm^2^ (range 1.1–1.9). Transmitral mean diastolic pressure gradient was 5.4 ± 2.7 mmHg (range 2–7). Estimated mean of the systolic pulmonary artery pressure was 40.1 ± 8.4 mmHg (range 25–45).

Mitral regurgitation was mild in three patients while it was trivial or none in the rest. Tricuspid regurgitation was graded as mild in four patients and minimal in the rest. All the patients were on monthly benzathine penicillin prophylaxis against recurrence of rheumatic fever. None was on diuretics or any other cardiac medications or has needed further intervention.

## Discussion

Currently the Inoue balloon technique is the standard technique for mitral valve dilation for treatment of mitral stenosis due to rheumatic heart disease or calcific mitral stenosis. The technique we describe here does not compare to the Inoue balloon technique in terms of ease and safety of operation. We do not imply that this technique is an alternative to the Inoue balloon under circumstances where the Inoue balloon is available and the operator is well versed with the technique. There is no doubt that the Inoue balloon is superior, if it is available and the operator is experienced with it.

Our technique is actually a modification of older singleballoon techniques used for the treatment of mitral stenosis.^20^,^21^ Compared to other single-balloon techniques, the Nucleus balloon offers the advantage of asymmetric inflation of both extremities before the central part of the balloon, thus ensuring some degree of stabilisation over the stenotic orifice.

Our technique is significantly different from that in which the Nucleus balloon has been used, in that it avoids placing a stiff wire in the left ventricle, decreasing the risk of ventricular arrhythmia, or hypotension from mitral interference. The use of a very floppy Terumo wire in our technique preserves mitral valve function until the arteriovenous loop is pulled for some seconds during balloon inflation. Furthermore, the risk of apical left ventricular rupture associated with the double balloon and other similar techniques is less likely to be a problem with our technique.

Establishment of an arteriovenous loop offers better balloon stability and can potentially be used with any other type of balloon available, especially in resource-limited settings. We felt that stabilising the balloon in that manner would be a particular advantage in our relatively young and small population of patients.

Indeed the procedure was adopted in our first patient, after initial inflation of the Nucleus balloon over a stiff wire placed at the left ventricular apex proved unsuccessful, the balloon being pushed back to the left atrium. The concern that applying tension on both ends of the Terumo wire may result in aortic injury may be overcome by placing a catheter over the wire, although we have not done this in the patients treated thus far. We used small balloons to dilate the septal puncture. Although there may be a theoretical risk of creating an iatrogenic atrial septal defect through the hole, this did not occur in any of our patients.

In initial publications describing single&ndash;balloon over&ndash;thewire procedures,^18^ Lock et al. positioned the exchange wire in the descending aorta, thus increasing support and stability for the balloon. The addition of the arteriovenous loop increases stability by pulling both ends of the wire, while using a softer guide wire. Although techniques using arteriovenous loops have been described previously,^19^,^22^ they never gained widespread acceptance, either because the wire was snared inside the left ventricle,^19^ or because the balloon was advanced through the arterial end of the loop.^22^ With the current catheters, guide wires and snares available, our technique is definitely much more straightforward than the originally proposed variants.

This technique may be considered a good option in resourcelimited settings where the Inoue balloon is not always an available option. Compared with the Inoue balloon, the total cost of the Nucleus balloon and its associated hardware is significantly less. Besides, the Nucleus balloon is easier to clean and resterilise as it has a single layer, compared to cleaning the Inoue balloon. It can be reused multiple times, offering a significant cost advantage in resource-limited settings such as ours. This technique may also be easier for use in children by paediatric interventionists who may not be familiar with the Inoue balloon technique but frequently use arteriovenous loops for other interventions.

One of our patients developed a tear in the anterior mitral valve leaflet and underwent valve replacement surgery. This complication may not be associated specifically with the described technique and could potentially occur with the Inoue balloon and other techniques. In fact, when inspected by the surgeon, the valve appeared too dysplastic to attempt repair. However, our technique carries a potential complication of inflating the balloon partially in the left ventricular outflow tract, thus avulsing the sub-valvar mitral apparatus. Great care has to be taken to not fully inflate the balloon if it seems to engage partially in the outflow tract during gentle initial inflation.

Although our patient population was small, the outcomes achieved in terms of increase in mitral valve area and reduction of mean transmitral diastolic gradient were comparable to those obtained with the Inoue balloon and other techniques.^21^,^23^ Estimated pulmonary artery pressure also dropped significantly. These outcomes were maintained on follow up at close to two years. Except for one patient who had an anterior leaflet tear leading to severe mitral regurgitation, the degree of mitral regurgitation was mild or less in all cases at the last follow up.

A limitation is that the number of patients in our study was relatively small. Furthermore, we did not compare our technique head to head with other techniques; it was based rather on a literature review.

## Conclusion

The Inoue balloon is not usually available in our centre as we get most of our consumables on donation. Our modified Nucleus balloon technique for mitral valve dilation in patients with mitral stenosis is effective and safe. The technique differs from other over-the-wire balloon techniques described in the past in that it avoids placing a stiff wire in the left ventricle, avoiding the risk of ventricular arrhythmia. It also offers better balloon stability and control owing to the arteriovenous loop. This technique can potentially be used with any other balloon available and may be easier for use by paediatric interventionists who might not be familiar with the Inoue balloon technique. TEE guidance is very useful to avoid the potential risk of inflating the balloon in the left ventricular outflow tract or through the sub-valvar apparatus. The Nucleus balloon can also be resterilised and used multiple times.
